# Changes in occurrence and management of laryngeal fractures at the Helsinki University Hospital during 25 years

**DOI:** 10.1007/s00405-023-08298-x

**Published:** 2023-11-03

**Authors:** Riikka E. Mäkitie, Kristofer Nyman, Taru Ilmarinen, Laura Tapiovaara

**Affiliations:** 1 Department of Otorhinolaryngology–Head and Neck Surgery, Head and Neck Center, Helsinki University Hospital and University of Helsinki, Kasarmikatu 11–13, FI-00029 Helsinki, Finland; 2 Clinicum, Faculty of Medicine, University of Helsinki, Helsinki, Finland; 3 Radiology, HUS Diagnostic Center, Helsinki University Hospital and University of Helsinki, Helsinki, Finland

**Keywords:** Larynx, Laryngeal fracture, Neck trauma, ENT, Tracheostomy

## Abstract

**Purpose:**

Laryngeal fracture is a rare but potentially life-threatening trauma. Fractures vary from mild to dislocated and extensive with risk of severe complications. This study investigated the occurrence, clinical characteristics and management of laryngeal fractures in the last 15 years.

**Material and methods:**

A retrospective population-based cohort study reviewing all laryngeal fractures at the Helsinki University Hospital in 2005–2019. Patient records and imaging studies were systematically reviewed for mode of injury, fracture type, secondary complications, treatment modality, possible airway management, length of stay, and mortality. Results were compared with corresponding data from 1995 to 2004.

**Results:**

Overall 80 fracture patients were recorded (5.3/year); 79% were men and mean age was 42 years (range 18–78). Altogether 91% were closed and 9% open. While unintentional traumas were most common (54%), an increasing proportion were from intentional injury (10%) or Schaefer Gr IV in severity (35%). Altogether 46% had compromised airway and 21% needed airway intervention; airway narrowing was more common with cricoid (*p* = 0.042) and multiple fractures (*p* = 0.07) and correlated positively with amount of dislocation (*p* = 0.001) and number of fracture lines (*p* = 0.006). Surgery was performed for 33%, of which 46% were Schaefer Gr IV and 62% from intentional trauma. Mortality was 1.4%.

**Conclusions:**

Deliberate and violence-related laryngeal fractures have increased. These often result in more extensive injuries predisposing to compromised airway and requiring surgical intervention and longer treatment. Most fractures are still treated conservatively with good long-term outcomes. An observation period of 24 h is recommended to detect any delayed complications. Mortality remains low.

## Introduction

Laryngeal trauma is a rare but potentially life-threatening trauma. Fractures of the laryngeal cartilages can result from both blunt and penetrating forces and vary in severity from mild and nondisplaced to dislocated and extensive with risk of severing adjacent structures and leading to severe complications [1–3]. Especially displaced fractures and/or in the presence of secondary complications, patients may need immediate or late-phase surgical intervention to maintain sufficient airway and restore breathing, vocal and swallowing functions [4].

Prior studies have reported laryngeal fractures to have an incidence of 1:14,000 and 1:30,000 of emergency visits and mortality of 1% [5, 6]. However, due to the rarity of such trauma, cohorts are often small, collected over a long time period and variable depending on the reporting center and their patient population; most recent studies are the United States reporting cohorts of 40–60 cases per 16–18-year study periods [2, 7, 8]. The latest report from Finland is an epidemiological study from our clinic in 2008 [4]. The study reported altogether 33 fractures between the years 1995 and 2004, with a higher prevalence in males (85%) and young adults (median age 34 years). Blunt force was the most common cause (97%), 61% were treated conservatively, 24% needed tracheostomy and 21% surgical intervention. Mortality was 0%.

Hence, and in continuance, we set out to investigate all laryngeal fractures treated at our clinic in 2005–2019. We aimed to evaluate the occurrence, causes, clinical characteristics and line of management of laryngeal traumas in the last 15 years, and secondly to compare these findings to corresponding data from the prior 10 years. Also, as laryngeal fractures still lack a standard protocol of care due to their relative rarity, we sought to outline the specific characteristics predicting delayed complications and need for surgical intervention.

## Material and methods

### Cohort

The cohort was composed by reviewing all laryngeal fractures at the Helsinki University Hospital (referral area 1.6 M) between 2005 and 2019. Patients were retrospectively collected from the patient record system using ICD-10 codes. To avoid selection bias, we scouted for several ICD-10 codes related to laryngeal trauma: S10.0 Contusion of throat, S11.0 Open wound of larynx and trachea, S11.2 Open wound of pharynx and cervical esophagus, S11.7 Multiple open wounds of neck, S12.8 Fracture of other parts of neck, S12.9 Fracture of neck, unspecified, S17.0 Crushing injury of larynx and trachea. Data on overall patient visits at the ENT emergency department were available for the years 2012 to 2019 and used to evaluate for change in patient volume and its impact on occurrence of trauma.

An institutional research permission was granted at the Helsinki University Hospital (46/2022).

### Clinical data

Patient records were systematically reviewed for patients’ sex and age, symptoms and findings on initial clinical examination, mode of injury, type, location and characteristics of fracture, secondary complications, delay to care, treatment modality, need for airway management or other surgical interventions, length of stay, long-term follow-up and mortality. Secondary factors, such as alcohol or substance abuse, were inconsistently recorded and therefore disregarded.

### Imaging data

Computed tomography (CT) imaging data were available for 92% (74/80) of the patients. Due to the retrospective study design, up to 12 CT devices from three vendors (GE, Siemens, and Toshiba) in ten hospitals were used. Slice thickness ranged from 0.6 mm to 3.0 mm and was 1.25 mm or less in 71% (57/80) of the studies. Intravenous iodine contrast agent was used in 23% (18/80) of the cases.

CT studies were systematically re-reviewed for mode of injury, type, number and location of fractures and possible secondary complications, such as soft tissue hematoma or emphysema, and airway narrowing. Maximum dislocation of the fractures was measured. Calcification of the cricoid and thyroid cartilages were visually estimated on a four-grade scale (0–3) depending on the rate of calcification (0–25%, 25–50%, 50–75%, and 75–100%). Evaluation was performed using both bone and soft tissue algorithm CT images and based on subjective radiologist impression after reviewing all images of the particular cartilage. All images were reviewed by an experienced head and neck radiologist (KN), who was blinded to the patients’ information and clinical status. Image analysis was performed using standard radiologic workstation and software (Siemens Syngo Plaza VB30D). Multiplanar reconstructions and both soft tissue and bone algorithm images were used when possible. All laryngeal injuries were graded according to the Schaefer classification scheme [9], which categorizes laryngeal injuries according to severity, ranging from minor endolaryngeal trauma with no fractures to severe injuries with multiple and unstable fractures to, finally, complete laryngotracheal separation.

### Statistical analyses

Data are presented as mean; ranges are given when suitable. Normality of data was assessed using Kolmogorov–Smirnov and Shapiro–Wilk and visually using histograms. Differences and correlations between parameters and subgroups were assessed using Mann–Whitney *U* test and Spearman correlation coefficients (SPSS Statistics 25; IBM Corporation, Armonk, NY, USA). A *p*-value < 0.050 was considered statistically significant.

## Results

**Fig. 1:**
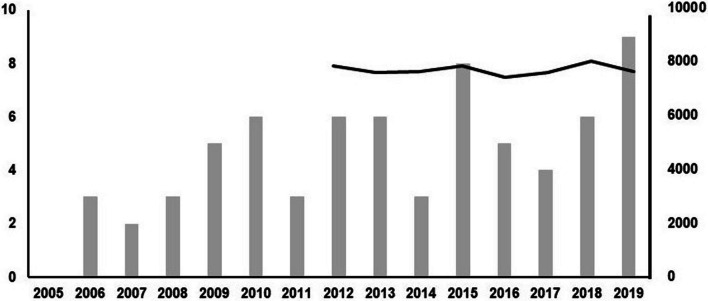
Annual occurrence of laryngeal fracture patients at the Helsinki University Hospital. Data collected over 15 consecutive years between 2005 and 2019. Data on overall patient visits at the ENT emergency department is shown for years 2012–2019 in solid line. Data show a steady rise in fracture cases during the recent years while overall patient volume has remained constant

In total 80 fracture patients were recorded during the 15-year study interval (5.3/year), showing a steady increase and a general increase of 60% compared to the prior ten years (Fig. 1; Table 1) [4]. This is despite the number of overall emergency department patient visits having remained constant: 7850 vs 7641 patients in 2012 vs 2019, respectively (Fig. 1). Of all fractures, six were diagnosed only during surgical exploration or by other means, and the rest (*N* = 74) from CT scans.

**Table 1:** Cohort demographics and fracture characteristics in two cohorts of laryngeal fracture patients

	Number (%)	
	1995–2004*	2005–2019
*Patients*		
Total	33	80
Males	28 (85)	63 (78.8)
Females	5 (15)	17 (21.2)
Mean age (years)	34 (median)	42.1
Males	N/A	41.6
Females	N/A	43.8
Schaefer-Fuhrman group**		
II	16 (48.5)	19 (23.4)
III	13 (39.4)	33 (41.3)
IV	4 (12.1)	28 (35.0)
V	0 (0.0)	0 (0.0)
*Fractures*		
Total	33	100
Thyroid	21 (65.6)	70 (70.0)
Cricoid	6 (18.2)	23 (23.0)
Hyoid	N/A	7 (7.0)
Multiple	6 (18.2)	19 (19.0)
*Management*		
Conservative	20 (61.0)	54 (67.5)
Airway management***	8 (24.0)	18 (22.5)
Surgical intervention	7 (21.0)	26 (32.5)

^*^As published by Juutilainen M et al (2008) Laryngeal fractures: clinical findings and considerations on suboptimal outcome. Acta Otolaryngol 128:213–218

^**^According to Schaefer SD (1992) The acute management of external laryngeal trauma. Arch Otolaryngol Head Neck Surg 118:598–604; and Fuhrman GM, Stieg FH 3rd, Buerk CA (1990) Blunt laryngeal trauma: classification and management protocol. J Trauma 30:87–92

^***^Intubation and/or tracheostomy

Overall mean age was 42.1 years (Table 1). Of the 80 patients, 63 were male (78.8%) with a mean age of 41.6 years (range 18–68). Women (*N* = 17) were slightly older with a mean age of 43.8 years (range 20–78 years), although the age difference was not statistically significant (*p* = 0.920). Patients' ethnicities were not recorded but are assumed to be representative of the Greater Helsinki area population.

### Clinical characteristics

**Fig. 2:**
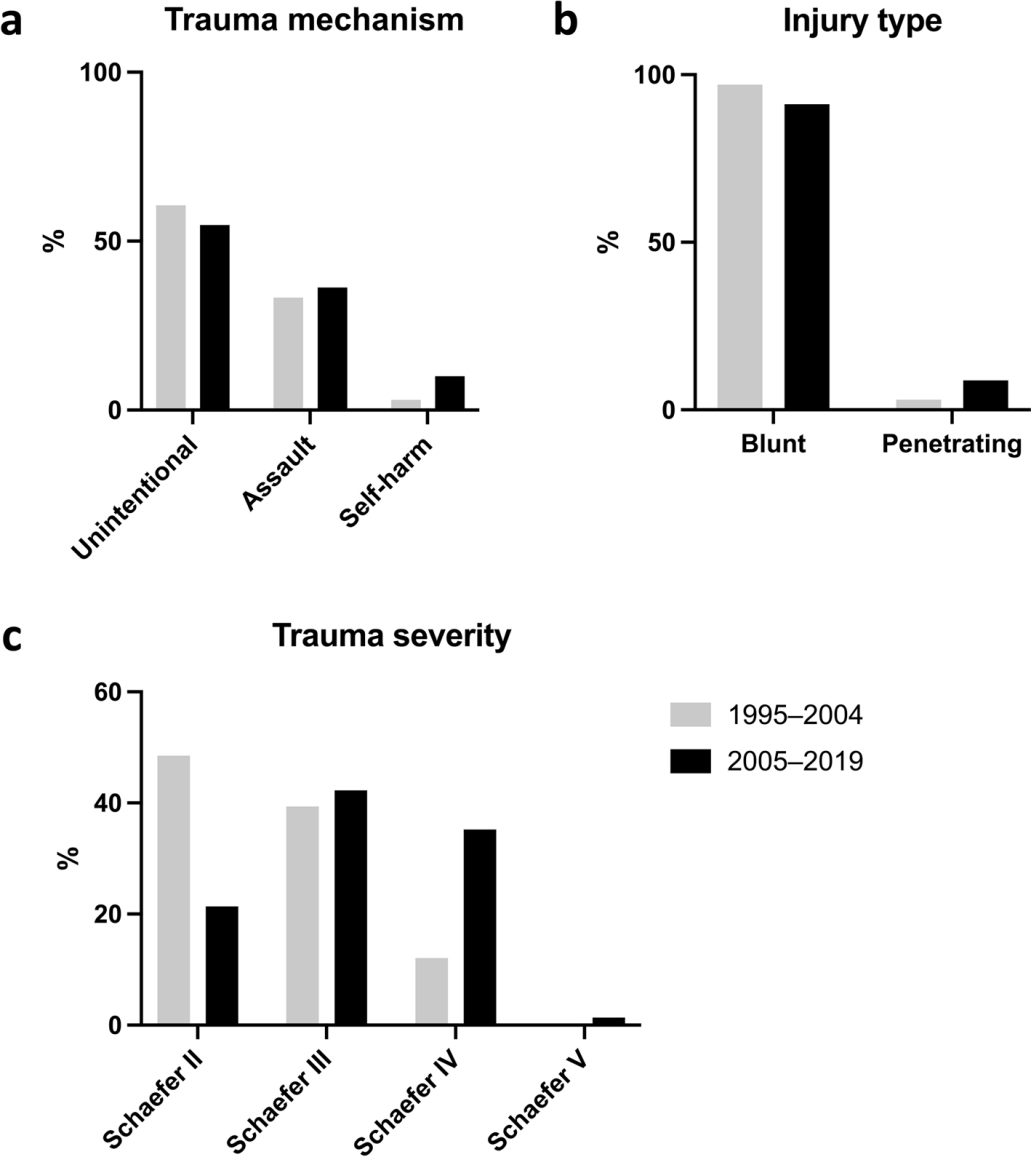
**A** Types of laryngeal fractures. Cohorts collected over two time points: 1995–2004 (gray) and 2005–2019 (black). Data show an increase in assault (33 vs 36%) and self-harm-related (3 vs 10%) traumas. **B** Injury types. Data show an increase in penetrating trauma from 3 to 9%. **C** Schaefer classification of laryngeal traumas. Data from altogether 33 patients from 1995–2004 (gray) and 80 patients from 2005–2019 (black). Data show an increase in more severe trauma (Schaefer Gr III and IV) in the recent years

Most of the fractures (*N* = 43, 53.8%) resulted from unintentional traumas, of which sports-related incidents accounted for the majority (*N* = 19, 44%, 23.8% of total; ice hockey being unequivocally most common), followed by a fall (*N* = 13, 30.2%, 16.3% of total) and motor vehicle incidents (*N* = 4, 9.3%, 5.0% of total) (Fig. 2a; Table 2). Physical assault was the second most common cause (*N* = 29, 36.3%), typically from suffocation (*N* = 21, 72.4%, 26.3% of total) and 26/29 in males. Eight (10%) resulted from deliberate self-harm, either from stabbing (*N* = 6) or suffocation (*N* = 2); 6/8 in males. Patients with assault or self-inflicted trauma tended to be younger than those with unintentional trauma (39.3 (*p* = 0.185) and 37.8 (*p* = 0.351) years, respectively, vs 44.8 years).

**Table 2:** Trauma mechanisms in two cohorts of laryngeal fracture patients

	Number (%)	
	1995–2004*	2005–2019
*Injury type*		
Blunt/closed	32 (97.0)	73 (91.2)
Penetrating/open	1 (3.0)	7 (8.8)
*Trauma mechanism*		
Unintentional	20 (60.6)	43 (54.8)
Sports	13 (65.0)	19 (44.0)
Fall	3 (15.0)	13 (30.2)
Motor vehicle	2 (10.0)	4 (9.3)
Other	2 (10.0)	7 (8.8)
Assault	11 (33.3)	29 (36.3)
Suffocation	N/A	21 (72.4)
Hit	N/A	5 (17.2)
Kick	N/A	3 (10.3)
Intended self-harm	1 (3.0)	8 (10.0)
Stabbing	N/A	6 (75.0)
Suffocation	N/A	2 (25.0)

^*^As published by Juutilainen M, et al (2008) Laryngeal fractures: clinical findings and considerations on suboptimal outcome. Acta Otolaryngol 128:213–218

On average, patients sought medical care 16.7 h after incident (range 0–8 days; excluding one outlier 180 days), and 51 (63.8%) within the first 24 h. Delay to ENT care was typically short; 28 (35%) were evaluated within the first 24 h, 59 (73.8%) within the first three days and 70 (87.5%) within a week from incident. The longest delays were 38 and 180 days; a patient initially diagnosed and operated for cervical fractures following an incidental fall, who then developed dysphagia and dyspnea (needing tracheostomy on day 10) and was subsequently found to have a cricoid fracture, and a patient examined months later for persisting dysphonia, respectively.

All patients were clinically evaluated, and their symptoms and physical findings recorded. At first evaluation, 80% (*N* = 56) experienced dysphonia/hoarseness, 44.3% (*N* = 31) dysphagia, 24.3% (*N* = 17) dyspnea and 62.9% (*N* = 44) pain or discomfort around their neck or during swallowing. Dysphonia, dysphagia or pain are based on subjective or objective presence of each symptom; no systematic evaluation or grading system was used. All patients experienced at least one, 50% at least two and 28.6% at least three of these symptoms. Further, pharyngeal and esophageal injuries were assessed during ENT evaluations and from CT scans. Ten patients were sedated or unconscious at the time of first examination, preventing exact evaluation. Excluding cases with penetrating/open trauma, no clear link was found between symptoms and trauma mechanism or type and severity of fracture.

**Fig. 3:**
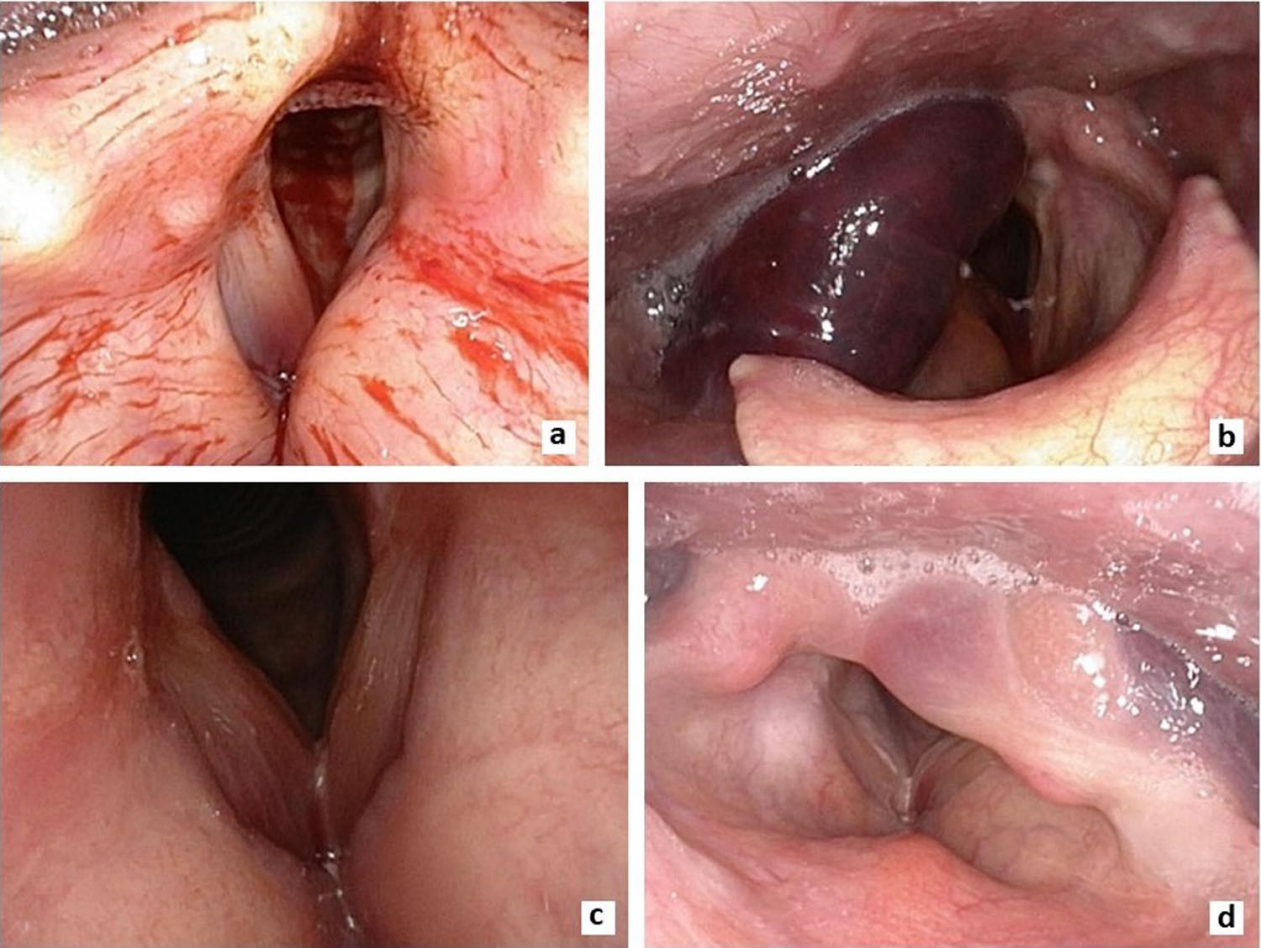
Endolaryngeal findings in patients with a laryngeal fracture

Altogether 73 (91.2%) of the fractures were closed and seven (8.8%) penetrating with open wounds (Table 1; Fig. 2). Of patients with closed fractures, 32.9% (*N* = 23) had swelling on their neck, 42.9% (*N* = 30) pain on palpation of neck and 87% (*N* = 61) noticeable changes in their larynx upon endoscopy (e.g. hematoma, swelling, paralysis, bleeding) (Fig. 3).

### Fracture characteristics

In total 100 fractures were recorded (from both CT scans and during surgery), of which 81 (81%) were isolated and the remaining 19 (19%) multi-site fractures. Patients with multiple fractures were statistically significantly older (mean age 50.2 vs 39.6 years, *p* = 0.012). Thyroid cartilage fracture was the most prevalent (*N* = 70, 70%) and involved in all multi-site fractures, typically together with the cricoid cartilage (73.7%). Cricoid fracture was the second most common (*N* = 24, 30%); nine of them were isolated (39.1%). No gender differences were noted but patients with a cricoid fracture were older than those without (38.6 vs 50.7 years, *p* = 0.002). Of the seven hyoid bone fractures, 6/7 were in conjunction with a thyroid cartilage fracture. One case of an isolated hyoid bone fracture was recorded: a male patient with deep wounds on his neck following self-inflicted stabbing.

**Fig. 4:**
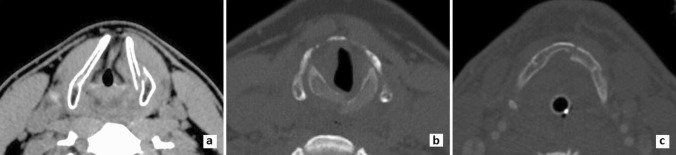
CT scans of patients with laryngeal traumas. **A** A displaced thyroid fracture in a 26-year-old male following suffocation on assault. **B** A displaced cricoid fracture in a 62-year-old female from an unintentional fall. **C** Hyoid fracture in a 56-year-old male in result of a motor vehicle incident

CT scans were available from 74 patients, and they were re-evaluated for closer details on fracture characteristics. Of the 65 thyroid cartilage fractures, 46 (70.1%) were unilateral, typically with a vertical lamina fracture line (77.3%) and with a mean dislocation of 1.4 mm (range 0–8 mm) (Fig. 4). The remaining 35.4% had a lamina fracture together with one or more cornu fractures. In total 42 had cornu fractures, of which 18 (42.9%) were isolated, 23 (54.8%) in the superior cornu and 14 (33%) in the inferior cornu, and nine (50%) from suffocation. Age did not correlate with the number of fracture lines (*p* = 0.750) or amount of dislocation (*p* = 0.677).

The 24 cricoid cartilage fractures were typically unilateral (*N* = 15, 62.5%) with a single fracture line (*N* = 15, 62.5%), involving the lamina (*N* = 13, 54.2%) and with a mean dislocation of 2.1 mm (range 0–5 mm) (Fig. 4). Age correlated positively with the number of fracture lines (*p* = 0.002) and with dislocation (*p* = 0.025).

Lastly, five hyoid bone fractures were observed in CT images, of which 4/5 were unilateral with one fracture line, involving the body (2/5) and/or a greater cornu (4/5) and with a mean dislocation of 0.11 mm (range 0–2 mm) (Fig. 4).

Most of the fractures were Schaefer Gr III in severity (*N* = 33, 41.3%), followed by Gr IV (*N* = 28, 35%) and Gr II (*N* = 19, 23.4%) (Fig. 2b; Table 1). Patients with Gr IV injuries tended to be slightly older (44.6 years) than Gr III (41.3 years) or Gr II (39.7 years) (*p* = 0.524 and 0.324, respectively).

Degree of cartilage calcification correlated positively with age for both thyroid (*p* = 0.004) and cricoid (*p* < 0.000) cartilages. Cartilage calcification for thyroid and cricoid was most commonly Gr 2 (40.5%) or Gr 1 (33.8%) and for cricoid Gr 2 (50%) or Gr 3 (23%). For thyroid cartilage, mean age for Gr 0–1 (*N* = 28) was 36.6 years and for Gr 2–3 (*N* = 46) 47.1 years (*p* = 0.002), and for cricoid cartilage 32.7 years (*N* = 20) and 47.0 years (*N* = 54) years, respectively (*p* < 0.001). Cartilage calcification did not correlate with the number of fracture lines or amount of dislocation for thyroid or cricoid.

For soft tissue and secondary complications observed in CT scans, 27 (33.8%) had partially and seven (8.8%) completely obstructed airway following a fracture. The degree of airway narrowing was significantly higher in the presence of a cricoid fracture (*p* = 0.045) and multiple fractures (*p* = 0.192) and correlated positively with amount of dislocation (*p* = 0.010) and number of fracture lines (0.006). Other common findings were soft tissue hematoma (in 77.5% of patients) or hematoma in the paraglottic space (61.3%), vocal cords (55%), false vocal cords (57.5%) or inside strap muscles (63.8%). Air emphysema was present in 15%. Vascular complications were rare; one patient had active bleeding (1.3%), likely from small veins, another had vertebral artery dissection following a motorcycle incident, and one suffocated patient showed a massive brain infarct three days after initial imaging, but CTA was not performed.

### Treatment and follow-up

In total 17 (21.3%) needed airway management: nine intubation (of which six were later tracheostomized for prolonged airway problems) and eight tracheostomy. For 13 (72.2%), intubation/tracheostomy was performed on the day of trauma and for the four other, on day 1, 5, 9 or 38. Duration of intubation/tracheostomy ranged from 2 to 28 days (mean 12.2 days), excluding one outlier 118 days, two for whom tracheostomy was permanent, and one who deceased before decannulation. Fourteen of the 17 had a thyroid fracture (82%) and four had multiple (23.5%). Schaefer grade, intended trauma and open injuries associated with need for airway management (*p* = 0.030, *p* < 0.001, *p* < 0.001, respectively), while patient sex or age or fractured cartilage did not.

**Fig. 5:**
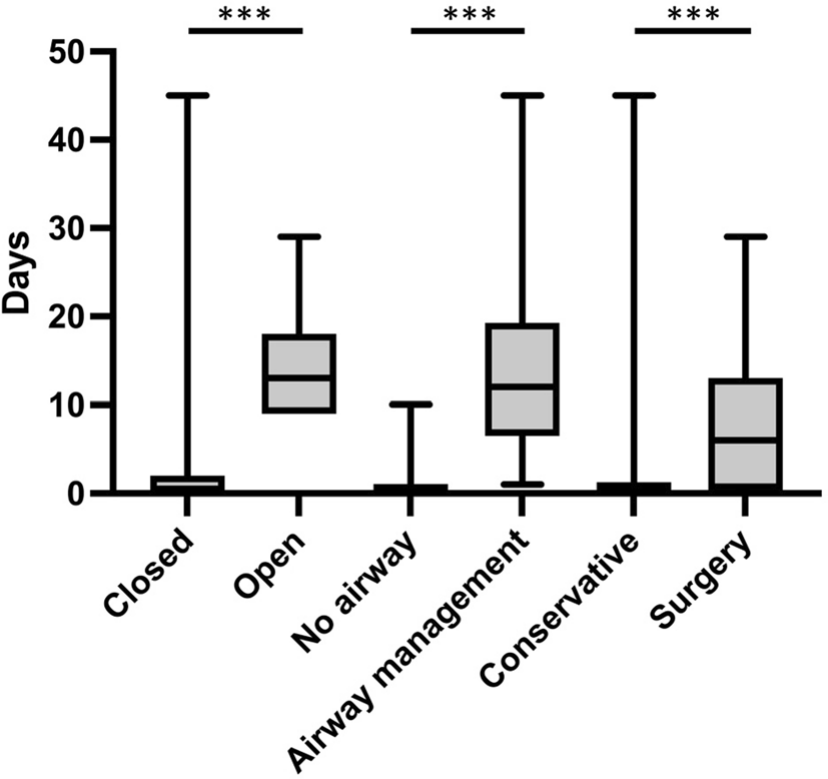
Length of hospital stays depending on type of trauma and modality of treatment. Data show a significant increase in treatment duration in patients with an open injury, compromised airway, and surgical intervention. ***p < 0.01

Of all patients, regardless of treatment, 45 (56.3%) were admitted and followed on the ENT ward, typically for a day to observe for any delayed airway complications, particularly advancing dyspnea or increasing neck or endolaryngeal swelling, prompting airway intervention. Patients were typically given oral steroids and PPI as pre-emptive measures for laryngeal swelling. Length of stay varied; range 0–45 days, mean 7.2 days and was significantly longer for patients with open injuries (median 0.5 vs 13 days, *p* < 0.000), Schafer Gr IV injuries (*p* = 0.002), and injuries needing airway management (median 0 vs 12 days, *p* < 0.001) or surgery (median 0 vs 6 days, *p* < 0.000) (Fig. 5).

In total 52 (65%) were treated conservatively. All of them had blunt traumas, 12 (23%) multiple fractures and three (5.8%) had needed airway management. After release from hospital, 38 were followed up (duration varied; range 5 days to 12 months) and the remaining 14 instructed to be in contact should long-term symptoms arise. Of the ones with an organized follow-up visit, 12 (31.6%) reported dissolved symptoms, 12 (31.6%) had mild dysphonia, five (13.2%) mild pain/laryngeal discomfort, and seven (18.4%) dysphonia not requiring late-stage surgical treatment.

The remaining 26 underwent surgery (22 males, 4 females, mean age 40.9 years, range 20–78 years), either in the form of initial surgical exploration with possible suture (*N* = 8), delayed exploration (*N* = 3) or delayed reduction and fixation (*N* = 15). Most of them had Schaefer Gr IV injury (Gr IV *N* = 12, 46.2%; Gr III *N* = 10, 38.5%; Gr II *N* = 4, 15.4%) and in 16/26 caused by intentional trauma (*p* = 0.002). Open fractures also associated with need for surgery (*p* < 0.001). Patient sex or age or fractured cartilage did not associate with tendency to operate. Immediate operations were often performed in a multidisciplinary fashion together with general, cardiothoracic and plastic surgeons. Elective surgeries were all performed by an experienced ENT surgeon and on average on day 6.5 (range 1–16). Fixation was done using titanium plates in all, in one also with a Montgomery prosthesis. One patient, despite initial surgical intervention, later died from his trauma (total mortality thus 1.4%).

## Discussion

Although still rare, laryngotracheal trauma can have devastating and long-term complications requiring extensive acute and/or delayed medical treatment. Here we report clinical and radiologic findings in 80 laryngeal fracture cases treated at our institution during 15 consecutive years. Our findings reveal an increase in overall occurrence of laryngeal fractures and a stark rise in intentional and severe injuries needing airway management, surgical intervention, and longer lengths of stay throughout the study years and, in comparison, to previous data from 1995 to 2004 [4]. Despite this, most can still be treated conservatively with good long-term outcomes, and mortality remains low.

Overall, laryngeal fractures have become more common, with an increase in average annual occurrence of 3.3 to 5.3 patients in the past 15 years. Majority (51%) are still caused by unintentional, blunt traumas and typically in 30- to 40-year-old men. These findings, as well as the location and the prevalence of multi-site fractures are congruent to earlier reports [2, 6, 10]. Sports are still a common cause of laryngeal and other head and neck injuries and consequently several rule modifications regarding protective gear have been made in the recent years. Considering the Finnish cohort, perhaps not surprisingly ice hockey was the most common cause; the Finnish Ice Hockey Association has implemented mandatory neck guards for all age groups just in the recent years, while still only recommended elsewhere in the World.

Despite this, interpersonal violence and intentional self-harm have significantly increased in the recent years, contributing to the higher prevalence of more severe injuries. In comparison to data from 1995 to 2004, most notable is the increase from 3 to 9% in penetrating traumas, from 3 to 10% in self-inflicted traumas, and from 12 to 35% in Schaefer Gr IV traumas—numbers comparable to ones reported by Olding et al. from a major trauma center in Central London with a high incidence of knife crime [10]. Such patients often require acute airway management (56%), multidisciplinary and surgical intervention, ICU monitoring and longer lengths of stay. In addition to the physical and psychological harm that may have long-lasting effects on an individual level, these also pose a greater burden on our health care system and socioeconomic well-being [10].

Laryngeal trauma, even in blunt and milder cases, results in symptoms prompting patients to seek acute medical care. Most patients (63.8%) were evaluated on the day of injury, followed by an, often immediate, ENT consultation. Congruent to earlier literature [6, 11, 12], dysphonia was the most common symptom, presenting in up to 80% of patients and possibly persisting for several months, though seldom requiring later surgical intervention. While external signs of laryngeal injury were rare, endoscopy revealed in over 87% laryngeal swelling, hematoma, vocal cord changes or airway narrowing. Interestingly no clear differences were found on vocal cord movement or endoscopic findings in thyroid or cricoid fractures. It is noteworthy, also, that a considerable proportion of patients can be completely asymptomatic at first, highlighting the importance of careful clinical examination and CT imaging [12].

Guidelines on acute- and late-phase treatment are variable, but most recommend a surveillance period of 24 h post-incident to observe for any immediate swelling and loss of airway patency [11, 12]. In our cohort, altogether 17 (21%) needed airway intervention. Factors associating with the need for airway management were Schaefer grade, intended trauma, and open injuries and as well as a cricoid cartilage fracture, especially ones dislocated or with multiple fracture lines, and multi-site fractures. Although most of them (72%) presented within the first 24 h, delayed airway problems did occur, supporting possibly extending the observation period in cases with extensive or unusual trauma. It is of utmost importance that initial evaluation carefully considers present or ensuing airway complications, and the treating emergency team acts on time to secure airway patency or organizes suitable surveillance with readiness to perform tracheotomy or cricothyroidotomy on standby, i.e. warranting stay on ENT ward. These are congruent to earlier findings of more extensive fractures (Schafer Gr III or higher) or intraluminal mucosal injuries requiring active airway management whereas Schaefer Gr II trauma can usually be managed conservatively with close monitoring for 24 h [6, 11]. The frequency of intervention varies across studies, ranging from 8.2% as reported by Moroco et al. to the significantly higher rate of 71.6% from a study by Bourdillon et al. [6, 13]. These are probably owing to the differences in study cohorts and period of data collection and echo the lack of clear consensus on initial airway management.

In the loss of airway patency due to laryngeal trauma, airway obstruction may sustain for several days and initial tracheostomy may be recommended. Of the nine initially intubated six were later tracheostomized. Also, time to decannulation varied greatly, but was on average 12 days and for three, considerably longer or permanent. These are congruent to reports from others: Jalisi et al. reported conversion of intubation to tracheostomy in 5/7 patients and average time to decannulation of 13 days [11], while a large review by Moroco et al. [6], consisting of 4134 patients, reported mean time of 10.4 days. Some have also argued against orotracheal intubation, as it may induce additional trauma such as cervical spine injuries, soft tissue edema, fracture dislocation, false lumen, laryngeal lacerations and hemorrhage, displaced laryngeal architecture and possibly compromised airway [6, 11, 14–16]. Schaefer et al. have suggested intubation when (1) larynx and trachea are clearly intact, (2) airway is visible on endoscopy, and (3) intubation can be performed by a highly experienced professional – in other cases intubation should be forgone and proceeded with tracheostomy [17].

In line with other reports [2, 7, 8, 17, 18], most fractures were in the thyroid cartilage and vertical in orientation, over half of the cricoid fractures were in conjunction with a thyroid fracture, in older patients and with greater dislocation and airway obstruction. Isolated hyoid bone fractures were scarce. Interestingly, we observed multiple isolated cornu fractures, typically in milder incidents and in half of the cases following suffocation. None of these patients underwent surgical fixation. Few studies have explored cornu fractures in laryngeal trauma, but they have been linked to strangulation and ligature hanging, likely owing to their anatomical position and relative weakness [19, 20]. While Knopke et al. described post-trauma pseudoarthrosis of the superior cornu resulting in odynophagia and relief of symptoms after surgical resection [21], other the literature remains sparse and their clinical relevance, need for surgical treatment and effect on patient outcome remain incompletely defined.

In total 33% required surgical management, which is an increase from the earlier study [4]. Frequency of surgical management varies across literature, with some reporting comparable numbers [2] while a recent review described up to 60% of patients undergoing operative treatment [6]. Excluding immediate neck exploration and surgical control of secondary complications, no clear consensus on the indications for and timing of surgical fracture fixation currently exist [17]. General consensus aligns with conservative follow up for Schaefer Gr I and II and surgical for the rest. In our cohort, Schaefer Gr III and IV comprised 85% of the operated cases; intended and open traumas were more likely to be operated on while patient- or fracture-related factors showed no clear association. Wang and colleagues made similar observations that while multi-site fractures and higher Schaefer Gr associated with need for airway and/or surgical management, no other associations (such as between mechanism of trauma or other fracture characteristics) could be found [2]. Most recommend fixation of displaced fractures to retain laryngeal framework and recent data suggests that earlier treatment leads to expedited recovery [17]. Excluding cases with evident need for control of airway patency or secondary complications, the decision to operate is likely due to multiple factors related to patient type and surgeon- or hospital-based customs.

Lastly, in case of acute penetrating or high-velocity blunt trauma, careful attention should be paid to possible vascular complications, as these are reported in up to 25% of penetrating neck trauma and can predict poorer outcome and carry devastating and life-threatening consequences [22–24]. Vascular trauma may be of penetrating origin causing vessel laceration, bleeding and haematoma, or blunt causing blunt cerebrovascular injuries. Indications for vascular imaging screening (CT angiography) usually follow the Denver criteria consisting of clinical signs, symptoms, and trauma mechanisms [25, 26]. Traditionally, the neck is divided into three anatomic zones to guide clinical management: (1) from the clavicle to cricoid cartilage, (2) from cricoid to the angle of the mandible, and (3) from the mandible to the base of skull. Zone 2 is the most commonly (80%) affected and surgically feasible zone, while the other two may require endovascular treatment. In our series, associated vascular trauma were rare and only observed in three patients. However, i.v. contrast was used only in 24% of the CT studies and CTA rarely done.

Our study has certain limitations, mainly concerning the relatively small cohort size and its retrospective nature. The variance in imaging modality and quality as well as detail and accuracy of clinical records may have introduced bias. Specifically, CT imaging has advanced in the recent years from a prior 3 to now 0.5 mm slice thickness, enabling precise and more accurate evaluation. Also, the use of ICD-10 codes for filtering may have excluded individuals with mild symptoms, no CT imaging and therefore no record of laryngeal injury. Furthermore, as follow-up visits were organized randomly on case-by-case basis and long-term symptoms not systematically evaluated, full effects of different fractures or outcomes of surgery cannot be assessed. Lastly, in the absence of a non-fracture control group, no definitive conclusions about the clinical findings and link to presence and/or severity of laryngeal fracture can be drawn. Despite these, given the rarity of laryngeal fractures, we consider our data unique and valuable.

In conclusion, timely evaluation of trauma and caution for possible secondary complications remains paramount in treating patients with blunt and penetrating laryngotracheal trauma. Interpersonal violence and intended self-harm have increased in the recent years, which is reflected in the number of patients with penetrating or extensive injuries requiring airway management and acute surgical intervention. Patients with a dislocated cricoid cartilage fracture, multi-site fractures or more severe trauma have greater tendency to lose airway patency and are recommended for close surveillance. Given that trauma severity predicts need for airway intervention and dictates acute and delayed treatment, obtaining an initial CT is of utmost importance. Most patients can still be treated conservatively with good long-term outcomes, and mortality remains low. Future research is warranted to further evaluate how specific fracture types and characteristics relate to different clinical symptoms and the need for surgical intervention.

## Data Availability

Data is available upon request from corresponding author.
